# Predictable phenotypic, but not karyotypic, evolution of populations with contrasting initial history

**DOI:** 10.1038/s41598-017-00968-1

**Published:** 2017-04-19

**Authors:** Pedro Simões, Inês Fragata, Sofia G. Seabra, Gonçalo S. Faria, Marta A. Santos, Michael R. Rose, Mauro Santos, Margarida Matos

**Affiliations:** 1 cE3c – Centre for Ecology, Evolution and Environmental Changes, Faculdade de Ciências, Universidade de Lisboa, Campo Grande, 1749-016 Lisboa Portugal; 2 Department of Ecology and Evolutionary Biology, University of California, Irvine, CA USA; 3 Departament de Genètica i de Microbiologia, Grup de Genòmica, Bioinformàtica i Biologia Evolutiva (GGBE), Universitat Autonòma de Barcelona, Barcelona, Spain; 4 Instituto Gulbenkian de Ciência, Oeiras, Portugal; 5 University of Saint Andrews, School of Biology, Saint Andrews, Scotland UK; 6 CEDOC – Centro de Estudos de Doenças Crónicas, Lisboa, Portugal

## Abstract

The relative impact of selection, chance and history will determine the predictability of evolution. There is a lack of empirical research on this subject, particularly in sexual organisms. Here we use experimental evolution to test the predictability of evolution. We analyse the real-time evolution of *Drosophila subobscura* populations derived from contrasting European latitudes placed in a novel laboratory environment. Each natural population was sampled twice within a three-year interval. We study evolutionary responses at both phenotypic (life-history, morphological and physiological traits) and karyotypic levels for around 30 generations of laboratory culture. Our results show (1) repeatable historical effects between years in the initial state, at both phenotypic and karyotypic levels; (2) predictable phenotypic evolution with general convergence except for body size; and (3) unpredictable karyotypic evolution. We conclude that the predictability of evolution is contingent on the trait and level of organization, highlighting the importance of studying multiple biological levels with respect to evolutionary patterns.

## Introduction

The relative importance of selection, genetic drift and historical effects determines how predictable evolution is, an issue in evolutionary biology that is still much debated^1–3^. In this context, experimental evolution under controlled conditions is a powerful tool because it allows the use of empirical evolutionary trajectories to test evolutionary repeatability and predictability, particularly when there are specific expectations about the outcome of such experimental evolution^1, 2, 4, 5^. Most of these experiments have been performed in viruses and microorganisms, and such studies have generally found repeatable phenotypic and genetic evolutionary responses (e.g. refs 6–11, see reviews refs 1, 2 and 12) with some qualifications. In fact, the repeatability of microbial evolution has been shown to depend on factors such as the population size^10^ and the environment tested^11^. Additionally, longer-term studies may lead to divergence, particularly due to the effects of unique mutations^13, 14^. In general, these studies have found that parallelism is greater at the phenotypic than at the molecular genetic level (e.g. refs 1, 2 and 15). The few experimental evolution studies that have tackled the repeatability of evolution at the molecular level in sexual organisms also support this expectation^16^.

The aforementioned experimental studies have analysed the evolution of populations starting from a common ancestral source. However, differences in genetic background between the founding populations used in studies of experimental evolution could generate historical contingencies that have an important impact on evolution, increasing the stochasticity of trajectories and outcomes^1, 13, 17–19^. This might be particularly relevant for fitness-related traits due to their highly polygenic basis and important non-additive effects^20^. Thus on the issue of how predictable is evolution it is important to consider experimental evolution among populations with contrasting origins.

Microbial experimental evolution research has shown that historical and chance effects on evolution can be particularly strong for traits more loosely related to fitness (e.g. ref. 18), with the impact of history being greater at the genetic than at the phenotypic level in bacteria^21^, yeast^22^, and viruses^23^. On the other hand, only a few experimental evolution studies have tested the impact of different genetic backgrounds in the evolution of sexual populations, mostly in *Drosophila* ^17, 19, 24–27^. These *Drosophila* studies also support the notion that historical effects on patterns of phenotypic evolution are somewhat limited, with most showing evidence of convergent evolution (e.g. refs 19, 24–29 but see ref. 17). One of the largest experiments is a reverse evolution study in *Drosophila melanogaster* ^19, 24^ where complete convergence to ancestral phenotypic values usually occurred after 50 generations^24^, with partial convergence at the molecular genetic level^30^. Nevertheless, the scope for historical effects in these studies is not high, as they have typically used laboratory populations that share a common ancestral founder (e.g. refs 19 and 25).

Latitudinal clines provide a natural source of highly differentiated populations with which to initialize parallel laboratory evolution experiments, in order to test for the impact of history on the predictability of evolutionary dynamics. A remarkable case of such variation comes from latitudinal clines of inversion polymorphisms, clines which suggest that these structures have a role in adaptation^31, 32^. Laboratory experimental studies in *Drosophila* on the evolution of inversion polymorphisms reinforce this hypothesis^33–36^.

*Drosophila subobscura* is an ideal organism with which to study the evolutionary forces affecting adaptation. It has repeatable clinal variation of chromosomal inversion frequencies and body size in Europe and the Americas^32, 37, 38^. Interestingly, inversion polymorphisms are changing worldwide as a result of global warming^32^. Furthermore, *D. subobscura* is amenable to laboratory culture and exhibits rapid phenotypic and genetic evolution in response to laboratory conditions^28, 29, 39^. Our team has been analysing the evolutionary dynamics of laboratory populations of *D. subobscura* starting from the first generations of their introduction to the laboratory from the wild. The laboratory is a novel environment to which such populations need to adapt^40^. Both biotic (e.g. densities, lack of competitors or predators, generation time) and abiotic (e.g. nutrients, temperature, light) conditions differ between nature and lab, imposing new challenges to naturally sampled populations. This evolutionary challenge allows us to characterize the dynamics of adaptation in sexual populations that are expected to have high initial standing genetic variation. In a previous study, Simões *et al*.^29^ showed that the phenotypic evolution of *D. subobscura* populations – sampled from nearby natural locations – in the laboratory environment may be contingent on the initial composition of the populations. There is evidence that this pattern of contingency might be due to stochastic events (such as sampling effects) that occur during the first generations in a new environment^4, 39^.

Recently, we have addressed the impact of historical effects on the evolutionary dynamics of initially differentiated *D. subobscura* populations in a novel laboratory environment^26, 27, 35^. In contrast to previous studies where we had sampled populations from nearby locations in Portugal^29^, we took advantage of the well-known European latitudinal cline in this species (see above), sampling wild populations from contrasting latitudes, which we know are genetically differentiated^26^. Our assumption is that these populations will have different degrees of adaptation to the novel conditions imposed by the laboratory, if they are locally adapted to the contrasting conditions in their natural locations (lower temperatures in the northern location, for instance). Moreover, contrasting genetic backgrounds - including variation in both the frequencies and the genetic content of chromosomal inversions as well as body size - may produce distinct evolutionary responses. In particular, the higher frequency of the so called ‘cold-adapted’ inversions in northern populations and ‘warm-adapted’ inversions in populations of more southern locations^32^ leads to the expectation that populations sampled from different locations along this latitudinal cline might adapt differently when placed in a novel laboratory environment featuring a constant, mild temperature, such as that imposed in our lab (18 °C). We found a clear initial impact of history at the start of the experiment, with quick convergence during laboratory adaptation for several life-history, physiological, and morphological traits^26, 27^ but not for chromosomal inversion frequencies^35^.

In the present study, we aim to test the predictability of laboratory evolution of *Drosophila subobscura* populations starting from different genetic and phenotypic standpoints as a result of prior historical differentiation in nature. We also want to address the predictability of evolution of populations that derive from the same naturally differentiated populations at different times. For this purpose, we sampled populations from the same two more-extremal European latitudes as those sampled three years before^26, 35^. We then compare the evolutionary responses among these four sets of populations, at both the phenotypic level (life-history, morphological, and physiological traits) and the karyotypic level, during their first ~30 generations of laboratory evolution. In addition to the expected initial genetic differentiation between populations from distinct latitudes, both sampling (founder) effects and temporal changes in the natural populations may affect the genetic composition of the starting populations derived from the same location in different years. This in turn may affect both the initial performance and the later evolutionary dynamics of the derived laboratory populations. We address the following questions. Is the impact of prior differences at the initial stages of experimental evolution in a novel environment repeatable across years of sampling? Can we predict evolutionary rates and outcomes even when populations differ in their initial state? Is the ability to predict evolutionary patterns dependent on the biological level studied?

## Results

### Phenotypic traits

#### Early Differentiation

**Figure 1:**
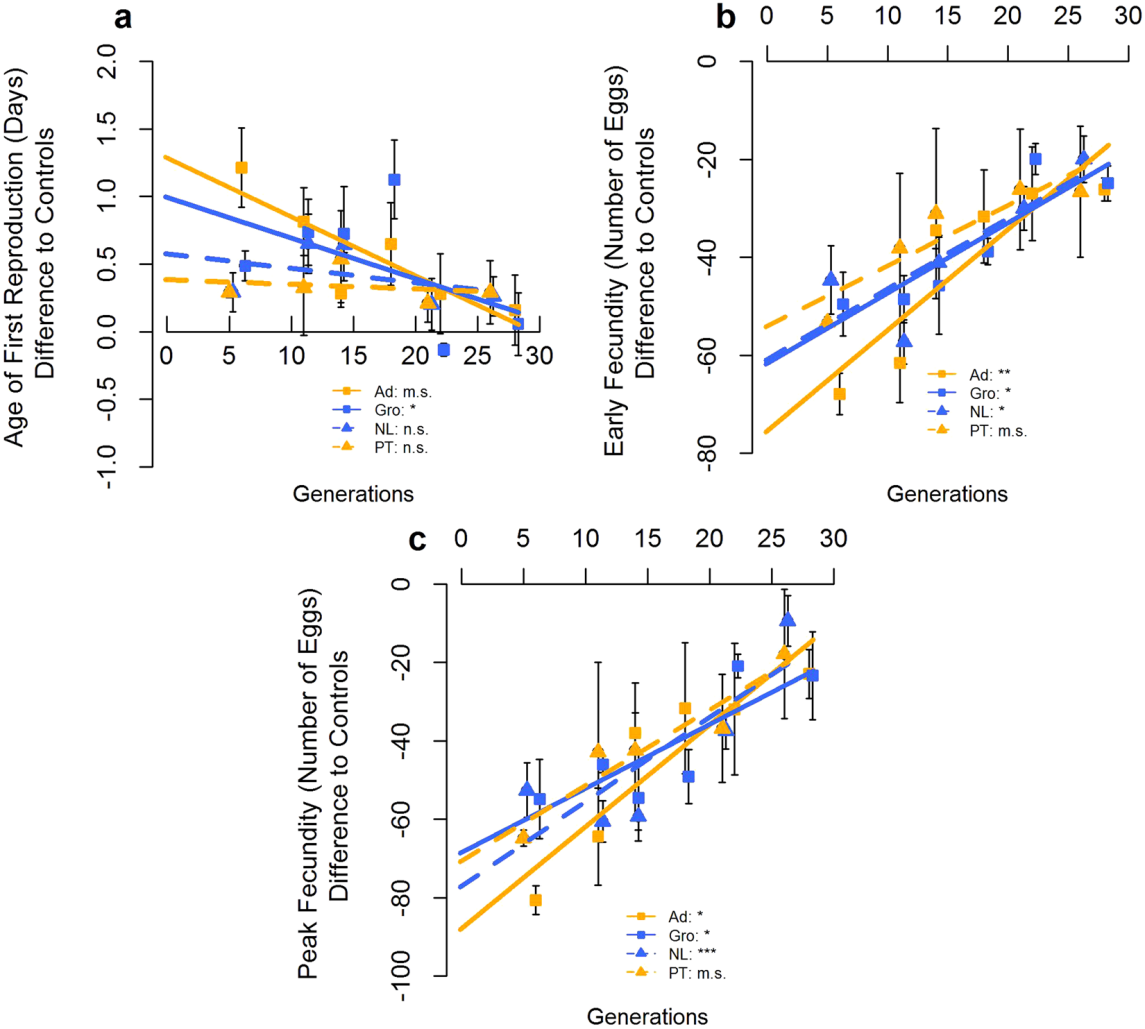
Evolutionary trajectories for the fecundity-related traits analysed. Age of First Reproduction (**a**), Early Fecundity (**b**) and Peak Fecundity (**c**) are presented for each foundation, as well as the corresponding linear regression models. Values shown correspond to differences between experimental populations and the average performance of the control (TA) populations assayed in synchrony at each generation. Error bars correspond to variation between replicate populations of each foundation. Significance levels of linear regression models: p > 0.1 n.s.; 0.1 > p > 0.05 m.s.; 0.05 > *p > 0.01; 0.01 > **p > 0.001; ***p < 0.001. Populations from Portugal (Ad and PT) – Orange; Populations from Netherlands (Gro and NL) – Blue; 2010 Populations (Ad and Gro) - Squares, solid line; 2013 Populations (PT and NL) – Triangles, dashed line.

**Figure 2:**
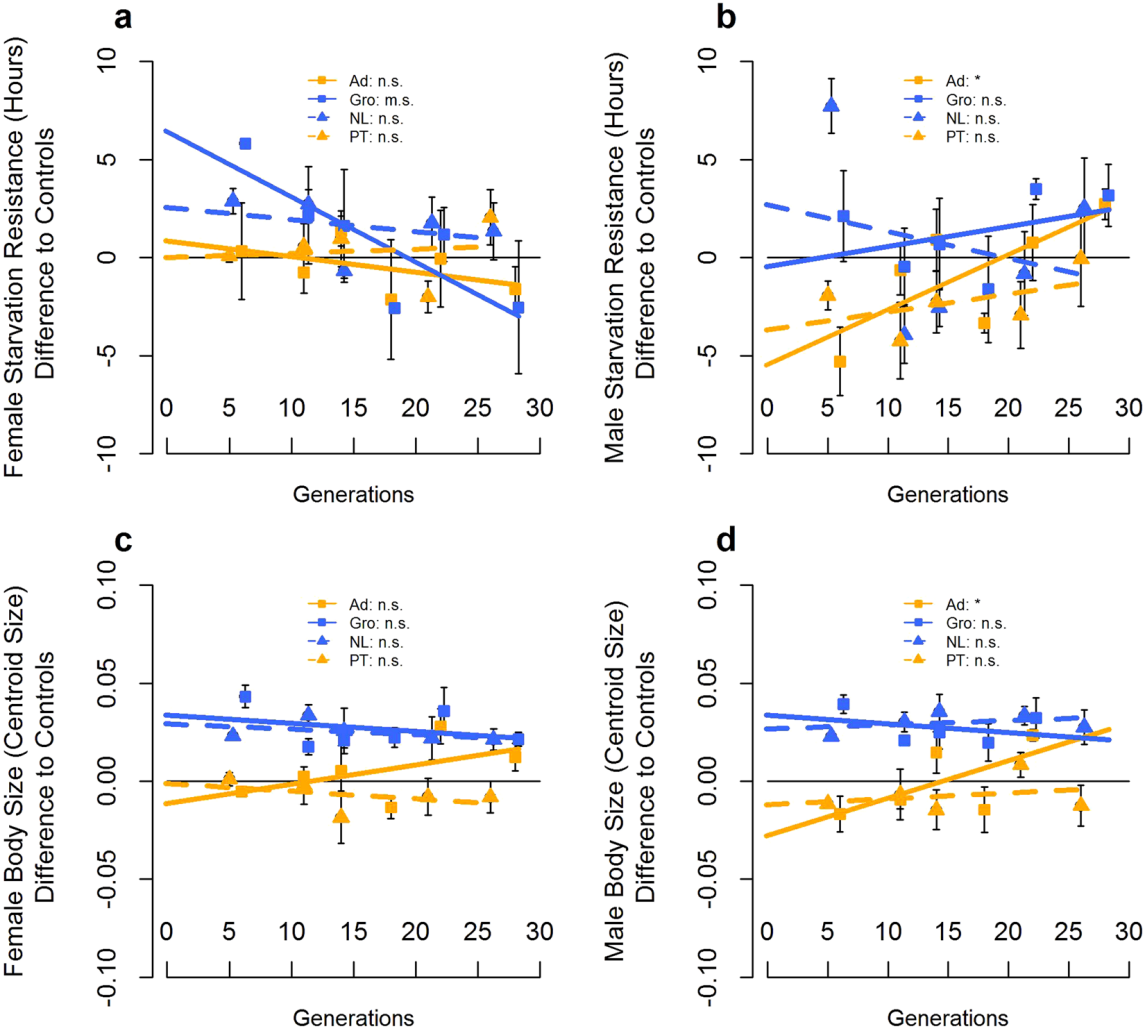
Evolutionary trajectories for starvation resistance and body size. Female Starvation Resistance (**a**), Male Starvation Resistance (**b**), Female Body Size (**c**) and Male Body Size (**d**) are presented for each foundation, as well as the corresponding linear regression models. See details in the legend of Fig. 1.

Populations founded in 2013 (PT from Portugal and NL from Netherlands) presented an overall better early performance for both age of first reproduction and male starvation resistance relative to those founded in 2010 (Ad from Adraga, Portugal and Gro from Groningen, Netherlands) (Year – Table 1; see Figs 1a and 2b). On the other hand, significant differences between locations, across years, were observed for all assayed traits except age of first reproduction, with populations from the Netherlands presenting higher performance (and bigger size) than their Portuguese counterparts (Location – Table 1, see Figs 1 and 2). In general, interactions between location and year were not significant, thus reflecting consistency of temporal patterns in the several traits analysed. The only exception was female body size, with a reduction of differences between the two locations in 2013 relative to those observed in 2010 (significant Location*Year term; Table 1; Fig. 2c). Comparisons between synchronously assayed populations from distinct locations (Ad *vs*. Gro; PT *vs*. NL) showed a significantly higher body size of both Gro and NL individuals compared to Ad and PT respectively, as well as a significantly higher starvation resistance of NL males and females compared to PT (Supplementary Table S1a). Additionally, the two 2010 foundations showed marginally significant differences in all fecundity and starvation resistance traits (Supplementary Table S1a). As expected, synchronously assayed control populations showed in general significantly better performance than the experimental populations for all fecundity related traits. Also, foundations from the Netherlands were always significantly bigger and showed consistently higher female and male starvation resistance than the controls (Fig. 2). Nevertheless, for starvation resistance, only the females from the 2010 Netherlands populations showed significantly better performance than the controls (Supplementary Table S1a).

**Table 1:** Analyses of differences in early differentiation (ANOVA) in phenotypic traits.

Model parameters	Age of First Reproduction	Early Fecundity	Peak Fecundity	Female Starvation Resistance	Male Starvation Resistance	Female Size	Male Size
Year	F_1,8_ = 10.292*	F_1,8_ = 3.453 n.s.	F_1,8.01_ = 1.923 n.s.	F_1,8.01_ = 1.628 n.s.	F_1,8_ = 7.379*	F_1,8.04_ = 4.127 m.s.	F_1,8.03_ = 1.069 n.s.
Location	F_1,8_ = 4.369 m.s.	F_1,8_ = 6.628*	F_1,8.01_ = 8.471*	F_1,8.01_ = 10.935*	F_1,8_ = 26.656***	F_1,8.04_ = 108.649***	F_1,8.03_ = 73.050***
Location*Year	F_1,8_ = 4.369 m.s.	F_1,8_ = 0.854 n.s.	F_1,8.01_ = 1.090 n.s.	F_1,8.01_ = 1.131 n.s.	F_1,8_ = 0.460 n.s.	F_1,8.04_ = 15.434**	F_1,8.03_ = 4.199 m.s.
Pop(Year*Location)	F_8,274_ = 0.959 n.s.	F_8,273_ = 1.869 m.s.	F_8,268_ = 1.851 m.s.	F_8,266_ = 2.364*	F_8,268_ = 2.827**	F_8,257_ = 1.058 n.s.	F_8,249_ = 2.044*

Note: significance levels: p > 0.1 n.s.; 0.1 > p > 0.05 m.s.; 0.05 > *p > 0.01; 0.01 > **p > 0.001; ***p < 0.001. Early differentiation analyses were performed with data from generation 6 of the 2010 foundations and generation 5 of the 2013 foundations. F statistic values are provided with the indication of degrees of freedom of the effect and error term.

After taking into account the effect of female body size on the other traits, differences between locations were no longer significant for early fecundity, peak fecundity, or female starvation resistance (Location – Supplementary Table S2a).

#### Evolutionary trajectories

All fecundity-related traits showed a significant evolutionary response in the laboratory across foundations, with increases in performance relative to the long-established, control populations (see Fig. 1a–c; Gen – Table 2, and Supplementary Table S3, for each foundation separately). A significant overall decline in female starvation resistance was observed through time across foundations (Gen – Table 2; Fig. 2a; see Supplementary Table S3). No overall evolutionary response across foundations was observed for either male starvation resistance or female body size (Gen – Table 2; Fig. 2b and c). For male body size, a significant increase was found for the 2010 Portuguese foundation (see Supplementary Table S3; Fig. 2d).

**Table 2:** Analyses of differences in evolutionary rate in phenotypic traits (ANCOVA) across generations.

Model parameters	Age of First Reproduction	Early Fecundity	Peak Fecundity	Female Starvation Resistance	Male Starvation Resistance	Female Size	Male Size
Gen	F_1,8.06_ = 21.107**	F_1,8.04_ = 113.46***	F_1,8.08_ = 141.02***	F_1,8.04_ = 7.847*	F_1,8.02_ = 0.931 n.s.	F_1,8.04_ = 0.000 n.s.	F_1,8.03_ = 8.639*
Pop(Year*Location)*Gen	F_8,1336_ = 0.846 n.s.	F_8,1335_ = 1.419 n.s.	F_8,1257_ = 0.983 n.s.	F_8,1253_ = 2.108*	F_8,1265_ = 3.990***	F_8,1182_ = 3.979***	F_8,1008_ = 3.007**
Location*Gen	F_1,8.06_ = 0.235 n.s.	F_1,8.04_ = 0.975 n.s.	F_1,8.08_ = 1.960 n.s.	F_1,8.04_ = 3.587 m.s.	F_1,8.02_ = 3.078 n.s.	F_1,8.04_ = 2.024 n.s.	F_1,8.03_ = 6.535*
Year*Gen	F_1,8.06_ = 10.610*	F_1,8.04_ = 2.220 n.s.	F_1,8.08_ = 0.435 n.s.	F_1,8.04_ = 3.971 m.s.	F_1,8.02_ = 3.711 m.s.	F_1,8.04_ = 1.741 n.s.	F_1,8.03_ = 0.057 n.s.
Location*Year*Gen	F_1,8.06_ = 1.474 n.s.	F_1,8.04_ = 1.568 n.s.	F_1,8.08_ = 2.444 n.s.	F_1,8.04_ = 1.295 n.s.	F_1,8.02_ = 0.031 n.s.	F_1,8.04_ = 3.615 m.s.	F_1,8.03_ = 3.113 n.s.

Note: significance levels: p > 0.1 n.s.; 0.1 > p > 0.05 m.s.; 0.05 > *p > 0.01; 0.01 > **p > 0.001; ***p < 0.001.

F statistic values are provided with the indication of degrees of freedom of the effect and error term.

Differences in the evolutionary response for age of first reproduction were observed between years of sampling, with 2010 foundations presenting a significantly higher response than those from 2013 (Year*Gen – Table 2, Fig. 1a), with the latter not showing a significant trend across generations (see Supplementary Table S3). Female body size differences may account in part for different evolutionary dynamics in age of first reproduction, as including female body size as covariate only produced a marginally significant Year*Gen interaction (Year*Gen – Supplementary Table S2b). In addition, significant differences in evolutionary rate between locations were obtained for male body size, with Portuguese foundations presenting a steeper positive response across generations relative to those from the Netherlands (significant Location*Gen term; Table 2, Fig. 2d) – although only the 2010 Portuguese foundation showed a significant trend (see Supplementary Table S3). The evolutionary response of starvation resistance presented marginally significant differences between years of sampling (Year*Gen term – Table 2). Taking into account the effect of body size (using only data until generations 21/22 for males – see methods), significant differences in the evolutionary rate for starvation resistance were found between years of sampling (Year*Gen – Supplementary Table S2b). This suggests that both male and female starvation resistance patterns are not due to the effect of body size.

Importantly, as found for early differentiation values, there was a general consistency in the differences in evolutionary dynamics between the two locations across years for all traits, as the location*year*generation interaction was non-significant, although marginally significant for female body size (Table 2). Also, synchronously evolving foundations (Ad *vs*. Gro; PT *vs*. NL) did not differ in their evolutionary rate for any trait analysed, despite marginally significant results for female and male body size and peak fecundity when comparing the 2010 foundations (see Supplementary Table S1b).

At the last generation assayed, no significant differences between foundations were found for most traits, both in the overall analysis comparing the two locations or years (Supplementary Table S4) or comparing the two locations sampled in each year (Supplementary Table S1c). The exceptions were female and male body size, which showed differences between locations (Location – Supplementary Table S4). For both traits, comparisons between locations within each year show that, for the 2013 foundations, males and females from the Netherlands still have significantly larger body size than their Portuguese counterparts, while for the 2010 foundations the initial differences in body size are no longer present at the final generation assayed (Supplementary Table S1c). Nevertheless, the Location*Year interaction term was not significant (Supplementary Table S4). Analysis between synchronously assayed experimental populations at generations 21/22 (previous to the last one analysed) presented in general similar results as those of the last generation assayed. The exception was female body size between the 2013 foundations which, contrary to generation 26, did not show significant differences at generation 21 (Supplementary Table S1d). A clear reduction of significant differences between experimental populations and controls was observed in the last generations assayed, relative to those initially observed (Supplementary Table S1a, c and d). Exceptions were early fecundity and body size. Early fecundity in both 2010 foundations and the 2013 Netherlands foundation was significantly lower than the controls at the last generation (Supplementary Table S1c and d). However, there was a clear reduction of differences through time indicating a convergence trend (Fig. 1). Also, females from the 2010 Netherlands foundation were significantly bigger than the controls in both generations 22 and 28, as in the initial generation (Supplementary Table S1a and c). This corresponded to a flat temporal trend (Fig. 2). A similar trend was observed for the 2013 Netherlands foundation, although with only marginally significant differences from the controls in the last generation. Male body size presented similar results, although no data was available for generation 28 of the 2010 foundations (Supplementary Table S1 and Fig. 2).

There was a highly significant dependence of the evolutionary rate on the early differentiation of populations, with higher initial differences to control leading to higher evolutionary responses. No significant differences between foundations were found for this association – see Supplementary Figure S1.

### Chromosomal Inversions

#### Early differentiation and subsequent evolution

**Figure 3:**
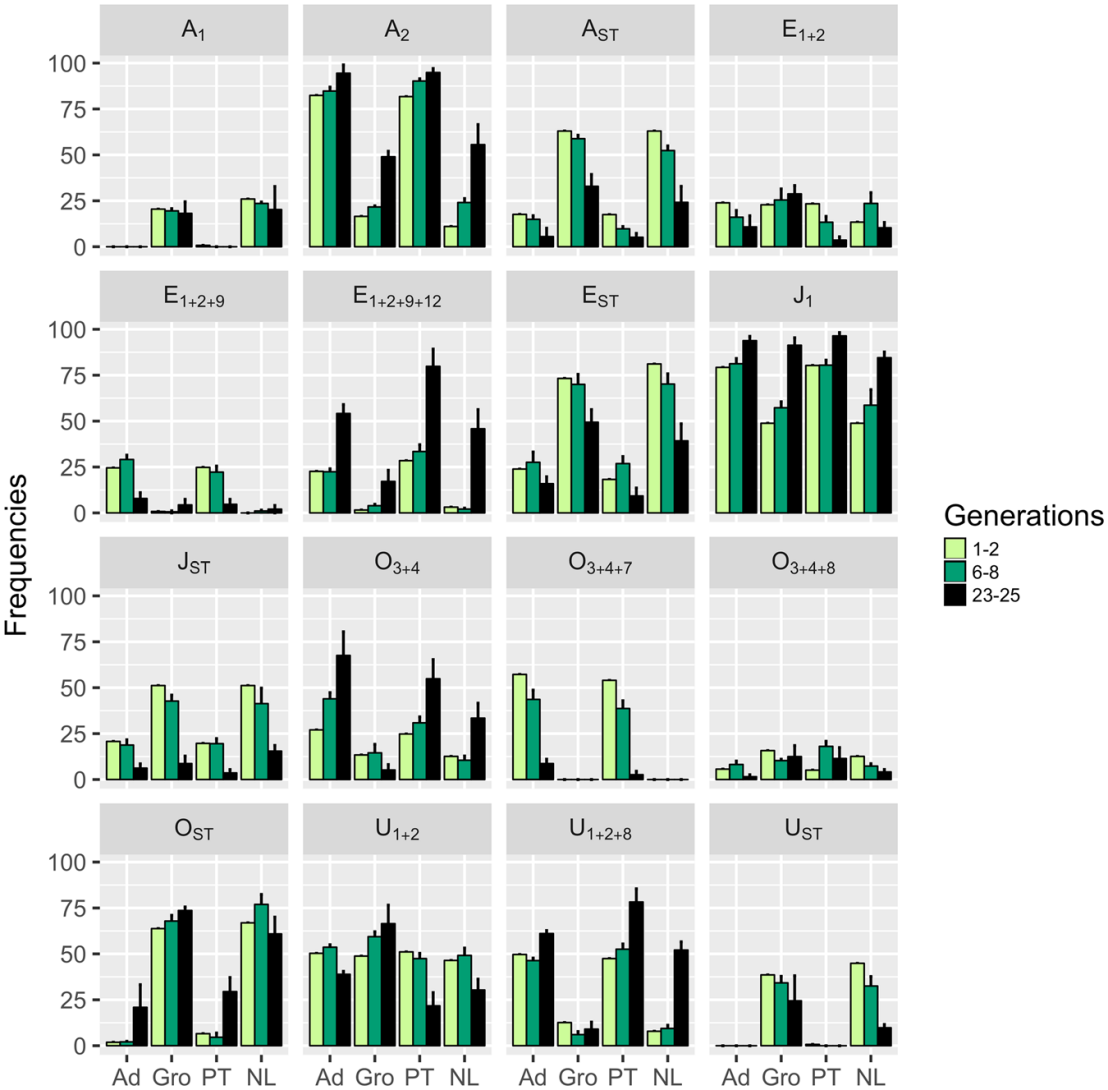
Frequency of chromosomal arrangements across generations and foundations. Error bars correspond to the standard error calculated from the differences among the three replicate populations of each foundation. See details in the Methods section.

Initial chromosomal inversion frequencies differed between locations, but not between years (Fig. 3; see Supplementary Table S5a). All foundations exhibited significant changes in inversion frequencies between the initial and final generations assayed (see Supplementary Table S5b). After 23/25 generations in the lab, differences between locations remained significant. However, these differences diminished significantly between the two 2013 foundations but not between those from 2010 – see Supplementary Table S5a. Differences between years were also significant at generations 23/25 – see Supplementary Table S5a.

A principal component analysis was performed on the transformed inversion frequencies (see Supplementary Figure S2). The first axis explained 38.49%, while the second axis explained 16.90% of the total variation. The inversions that made a greater contribution to variation in the first axis are clearly associated with clinal variation in nature (namely A_2_, U_1+2+8_, O_3+4_, E_1+2+9+12_, J_1_ *vs*. A_ST_, A_1_, E_ST_, O_ST_, U_ST_; see ref. 41). Combined analysis of both these axes shows a clear effect of geographical differentiation associated with nature and temporal changes due to evolution in the lab. The latter correspond to a pattern of reduction of initial differences between the 2013 foundations not observed for the 2010 foundations, in accordance with the genetic differentiation measures (see Supplementary Table S5).

#### Evolutionary dynamics of specific inversions

ANOVAs of the evolutionary dynamics of inversion frequencies showed some consistent patterns across foundations. Namely, there was a general decrease in frequency of A_ST_ (increase in A_2_), E_ST_, J_ST_ (increase in J_1_) and U_ST_ as well as an increase of E_1+2+9+12_. A consistent decrease of O_3+4+7_ frequency and an increase in E_1+2+9_ were also found in both Portuguese foundations (see Fig. 3 and Supplementary Table S6b). However, differences in the evolutionary dynamics were found between locations for several inversions (see Supplementary Table S6a). These dynamics were associated with either different rates of response (A_2_, E_ST_ and J_ST_), or with differences in the direction of the evolutionary changes (E_1+2_, O_3+4_, O_ST_ and U_1+2_) – see Fig. 3 and Supplementary Table S6. Importantly, such differences in direction between locations occurred mainly in the 2010 foundations (with the exception of O_ST_), causing divergence of inversion frequencies through time (see Fig. 3). Nevertheless, there were no significant changes across years in the differences in evolutionary dynamics between locations except for U_1+2+8_ (Year*Location - Supplementary Table S6a; see Fig. 3). Differences between years were only found for E_1+2+9+12_, U_1+2_ and U_1+2+8_ (Year term, Supplementary Table S6), with the two foundations from the Netherlands presenting the most striking contrasts – see Fig. 3.

According to the Cochran–Mantel–Haenszel (CMH) chi-squared test (see Supplementary Table S7), most inversions presented significant temporal changes during evolution in the lab. Exceptions were A_1_ in both foundations from the Netherlands (Gro and NL); O_3+4+8_ in Gro and in both 2013 foundations (PT and NL); E_1+2_ in the two 2010 foundations (Ad and Gro) and O_ST_, U_1+2+8_ and U_1+2_ in Gro (Supplementary Table S7).

In order to test for a significant deviation of inversion frequency dynamics relative to neutral expectations, simulations based on a neutral Wright-Fisher model (see details in the Methods section) were performed on the specific inversions identified using the CMH statistic. The expected frequencies generated by these simulations were then compared to the observed frequencies. To test for the foundations with inversion frequencies that significantly deviate from neutrality, the criterion used was that at least two of the three replicate populations (after FDR correction) presented significant results for a given inversion. Significant departures from neutral expectations were found for O_3+4+7_ (decrease) in both Portuguese foundations (Ad and PT); E_1+2+9+12_ (increase) in the 2013 foundations (PT and NL); O_3+4_ (increase) in Ad, J_ST_ (decrease) in Gro, and also A_ST_, E_ST_ (decrease) and A_2_, U_1+2+8_ (increase) in NL (see Supplementary Table S7). Overall, with the exception of O_3+4+7_ in the Portuguese foundations, inferred selection was not repeatable between years of foundation. Interestingly, NL populations exhibited the highest number of inversions possibly under selection (see above) with evolutionary changes in all these inversions moving them towards the values of the Portuguese foundations (see Fig. 3 and Supplementary Figure S2).

## Discussion

The initial state of populations has been shown to influence the subsequent dynamics and outcomes of experimental evolution^1, 19, 26, 29, 35, 39^. Here, by analysing populations of *Drosophila subobscura* derived from contrasting latitudes over multiple years, we found that geographical origin rather than between-year variation was more likely to produce disparate starting points for evolution. This indicates that variation in the founding populations – due to sampling effects or temporal variation in natural populations – is minor compared to the magnitude of historical differentiation among populations.

In general, we found repeatability across years in the magnitude of initial differences between populations from distinct locations. At the phenotypic level, an interesting exception is body size, which had higher differentiation between the 2010 foundations than between the 2013 ones. Similarly, for inversion frequencies early differentiation between locations was quite repeatable across years. Overall, our results point to a consistent impact of prior history on the initial stages of laboratory adaptation. In spite of this, there were temporal differences within locations, particularly for age of first reproduction and male starvation resistance. By contrast, there was general stability at the karyotypic level, which is quite interesting considering the evidence for temporal variation of inversion frequencies in natural populations of *Drosophila* ^31, 32^.

Overall, the impact of prior differentiation on laboratory evolutionary dynamics was consistent across years for most phenotypic traits. In general, a temporal reduction of differences was found, with newly-derived populations evolving toward the phenotypic values of the long established laboratory populations. This suggests the existence of one optimum to which traits values converge (see also ref. 26). Again, body size was an exception, exhibiting more contingent evolution depending on time of sampling. Actually, the evolutionary patterns for this trait could not be predicted between years, with a pattern of convergence obtained for the 2010 foundations but parallel evolution for the 2013 foundations. The greater contingency in the dynamics of this trait in comparison with fecundity might be explained by a weaker selective pressure and increased impact of random processes and/or evolutionary constraints on body size^42^. Nevertheless, this is not likely because, although body size is not itself a life-history trait, it is expected to have an important impact on fecundity^43, 44^. Though we do not have a clear explanation for this pattern, it might involve an interaction between founder effects and the different initial genetic background between populations, that derive from extremal latitudes of the body size European cline (ref. 38), particularly if genes less correlated with fitness are affected. Age of first reproduction, a trait more likely to be relevant in terms of fitness in our evolutionary scenario, showed differences in the evolutionary dynamics of populations from distinct years. However, this trait showed contrasting initial values between years (see above), particularly for the Portuguese foundations (see Fig. 1) and its evolutionary dynamics feature a clearly convergent pattern across all foundations. This suggests that for this trait convergence was in fact a predictable outcome.

In a previous study (Simões *et al*.^29^), we found more contingent evolution in starvation resistance – a physiological trait - than in fecundity, a trait more closely related to fitness (see refs 45 and 46), particularly in our environment (but see refs 47 and 48, for several examples of association between starvation resistance and longevity). Nevertheless, in that study, the variation in dynamics for starvation resistance between populations was relatively predictable when taking into account its initial performance, with general convergence among foundations. Here, we also observed a general tendency for a reduction of differences in starvation resistance between foundations, similar to the evolutionary patterns found for fecundity characters. The disparity of evolutionary patterns between starvation resistance and body size that we have found (see above) is perhaps counterintuitive, as a correlation between the two traits is expected^48^. Additionally, body size is more likely to be relevant to fitness than starvation resistance in the laboratory (see above). As such, our results do not point to a lower predictability of evolutionary trajectories in phenotypic traits less likely to be closely related to fitness (in our case starvation resistance), as might be expected based on previously published experimental evidence showing a higher impact of historical and chance effects on the evolution of such traits (e.g. refs 18 and 25).

In general, the temporal reduction of initial differences among populations for most traits indicates that those populations that are farther away from the value of the long-established populations are the ones that evolve fastest (Supplementary Figure S1). This explains why the evolutionary history of populations is erased quickly, as selection seems to be more effective the farther the populations are from the phenotypic values and genetic frequencies of populations that have long been cultured in our laboratory. It is possible that these patterns might result from contrasting levels of standing genetic variation present in the first generations after sampling from the wild, though these were not estimated. The similar dependence of evolutionary rate on early differentiation observed across foundations from different years and locations reinforces the predictability of phenotypic evolution in our study. These patterns have also been observed in other studies of phenotypic evolution^19, 28^, although those studies did not involve populations with the contrasting evolutionary histories of ours. It is important to note that our study covers 26–28 generations of evolution. The fact that we found a pattern of convergent evolution between newly founded experimental populations for several traits does not allow us to claim that longer-term studies will not eventually lead to later divergence. Prolonged observation of the evolutionary dynamics of these populations will also allow us to test whether later convergence occurs for body size, a trait still differentiated between the populations founded in 2013. Finally, adding more generations will be essential to determine whether the lack of full convergence that we detected, particularly for early fecundity and body size relative to longer-established populations, corresponds to a later convergence to the controls or to sustained differences, e.g. due to founder events or genetic drift (see also ref. 26).

Karyotypic evolution was not predictable across years, despite repeatable initial composition of inversions among populations derived from the same locations in different years. Thus, notwithstanding some common patterns, we could not predict the overall evolution of chromosomal inversion frequencies based on their initial composition. Several findings support our inference of unpredictability. Overall changes in inversion polymorphism indicated a trend of convergence for the 2013 populations, but this was not observed in 2010. Several inversions contributed to this pattern: E_1+2_, O_3+4_, U_1+2_ and U_1+2+8_. For these inversions, frequencies in the 2013 populations from Netherlands converged towards the values presented by both 2010 and 2013 Portuguese populations, while for the 2010 populations from Netherlands divergence was observed. These contrasting patterns may even lead, given sufficient time, to the fixation of different inversions between the laboratory populations of the 2010 foundations, but possibly not in the 2013 foundations. In addition, the temporal changes in inversion frequencies were in general larger for the populations founded in 2013. Finally, except for inversion O_3+4+7_ in the Portuguese samples, patterns of response to selection in inversions were not consistent between the two years studied, a finding which suggests that an interaction between selection and drift might have shaped the contrasting dynamics between years.

We previously^35^ put forward several hypotheses for the lack of convergence between the 2010 populations from Portugal and Netherlands for chromosomal inversions, in contrast with overall convergence at the phenotypic level. A simple explanation would be the lack of association between inversions and phenotypic traits. However, associations between inversion frequencies and several traits (morphological, physiological, and life-history) have been found in several species (e.g. see ref. 31 for a review). Particularly in *D. subobscura*, inversion polymorphisms have been linked to such traits as body size^49, 50^, thermotolerance^51^ and thermal preference^52^, mating success^53^ and viability^54^. These studies support the hypothesis that the contrasting patterns between inversions and the phenotypic traits are not due to a lack of association between them. Other hypotheses previously addressed^35^ were the possibility that different starting inversion frequencies and/or geographical variation in the genetic content of a given inversion affected the evolutionary dynamics. Here we show that populations starting with the same inversion frequencies as those studied by Fragata *et al*.^35^ show a convergent pattern. Thus it is unlikely that different starting inversion frequencies are responsible for the evolutionary dynamics of that study. Rather it suggests that temporal and spatial variation in the genetic content of inversions plays a major role in the evolution of inversion frequencies.

However, geographical variation in the genetic composition of inversions has not been observed in molecular studies in this species^55, 56^. Nevertheless, we inferred such differentiation in genetic content from temporal changes in the association between inversions and body size during experimental evolution of populations derived from the 2010 foundations^50^. Our findings differ from those of Inoue^57^, who reported general loss of inversion types in a controlled environment in *Drosophila melanogaster* populations. By contrast, we found that in our populations the inversion frequency changes are complex, with some inversions that are initially rare increasing consistently in frequency (see also ref. 34). However, these patterns were not predictable across years for some of those inversions (e.g. O_3+4_ and U_1+2+8_).

In this study, we show that the effects of history on the evolutionary dynamics of populations are not necessarily predictable at the karyotype level, despite being relatively predictable at the phenotypic level. It has been suggested that the predictability of evolution should be greater at higher levels of biological organization and ultimately also in terms of fitness itself^1, 15^. In general, evolution experiments in microorganisms have found surprisingly parallel evolutionary responses at the phenotypic and gene level^1, 2^, despite evidence of the impact of historical contingencies (e.g. ref. 7 and 13). On the other hand, higher unpredictability at the level of individual mutations was generally found (e.g. refs 8 and 12 but see refs 1 and 58). These results are in accordance with the notion that there are likely many more molecular combinations that can generate higher fitness than combinations of phenotypes that can. Therefore, parallelism should be higher at the phenotypic level (e.g. ref. 15). Very few studies have tackled these questions using organisms with abundant standing genetic variation (for an exception see refs. 24 and 30). It is expected that standing genetic variation will lead to more repeatable patterns than de novo mutations. There will be a finite number of pre-existing advantageous alleles of modest-to large effect that a new selection regime will favor, making it more likely they will be common across replicate populations^16^. Also, these alleles have probably already passed through a ‘selective filter’, thus increasing the probability of repeated outcomes^59^. Recent evolve & re-sequence studies in yeast and *Drosophila* support this expectation^16, 60–62^. Nevertheless, here we provide empirical support for the view that, in sexual populations as in asexual populations, evolution is more predictable for phenotypes than genotypes. It is important to note that the genetic changes we analyse here are at the karyotypic level. Future temporal analysis of genomic sequences during both initial and later generations will complement the genetic characterization of our populations.

## Methods

### Foundation and Maintenance of Laboratory Populations

*Drosophila subobscura* flies were collected in August 2010 from Adraga (Portugal) and Groningen (Netherlands), yielding two foundations in the laboratory that are called “Ad” and Gro”, respectively. The number of founding females was 234 for Adraga and 160 for Groningen. Two new collections from the same natural locations were made in August 2013, with 213 founding females from Adraga (Portugal) and 170 from Groningen (Netherlands). These gave rise to two new laboratory foundations, “PT” and “NL” respectively.

All populations were maintained under similar conditions^26^. Females were maintained as families during the first two generations and outbred populations were originated in generation three as described elsewhere. Foundations were three-fold replicated at generation 4. We will use the term “foundation” to refer to the set of three replicate populations derived from the same natural collection.

Three long established populations (TA – formerly “TW” populations – see ref. 28) were used as controls and assayed in synchrony with the experimental populations. These control populations were founded in 2001 from Adraga (Portugal) and maintained under the same laboratory conditions. When the 2010 and 2013 populations were introduced to the laboratory environment, these controls had been in the laboratory for 115 and 153 generations, respectively, and are thus most likely close to evolutionary equilibrium. This is corroborated by the absence of significant temporal trends for most traits (Supplementary Figure S3). In general, it is important to include these control populations in the assays so that we can take into account potential fluctuations in phenotypic traits due to environmental noise, as well as to remove possible spurious temporal patterns that may not relate with evolutionary trends (see below).

Populations were maintained with synchronous discrete generations of 28 days, reproduction near peak fecundity, 12 hours light:12 hours dark at 18 °C, and census sizes generally between 500 and 1200 individuals. Controlled density both for eggs and adults was sustained (see details in refs 26, 35 and 50).

### Phenotypic assays

For the 2010 populations, phenotypic assays were performed at generations 6, 11, 14, 18, 22 and 28 after introduction in the laboratory; assays of the 2013 populations were performed at generations 5, 11, 14, 21 and 26. Sample sizes per population, per assay, varied between 15 and 24 pairs of flies. Assayed flies were transferred daily and the number of eggs laid by each female was counted during the first 12 days. At the 12^th^ day the flies were transferred to agar medium and scored for starvation resistance. Male and female starvation resistances (RM and RF, respectively) were estimated as the number of hours until death (registered every 6 h after transfer to agar). Three fecundity-related traits were estimated: age of first reproduction (number of days between emergence and the first egg laying; A1R), early fecundity (total number of eggs laid during the first week of life; F1–7), and peak fecundity (total number of eggs laid between days 8 and 12; F8–12). Body size (BS) was measured for all assayed flies, with the exception of generation 28 for Ad and Gro for which no male measurements were available. As a proxy metric for this trait we used wing size, estimated by geometric morphometric analysis (see refs 26 and 50). Briefly, for the wing size measurements, *x* and *y* coordinates of 13 morphological wing landmarks were obtained^26^. From the original landmark coordinates, wing size was estimated as centroid size^63^. Our analyses used log-transformed centroid size values.

### Chromosomal Inversion Scoring

Chromosomal inversions were scored at generations 2, 6 and 25 in the 2010 populations and at generations 1, 8 and 23 in the 2013 populations. Three replicate populations were analysed per foundation, except at generations one and two, when the populations had not yet been replicated. Chromosomal inversions were determined by scoring one 3^rd^ instar larva originated by individually crossing males to virgin females from a marker homokaryotypic strain as described in refs 35 and 50. The number of individuals analysed per population varied between 65 and 159 males. Chromosomal arrangements were classified according to Kunze-Mühl & Müller^64^.

### Statistical methods

#### Phenotypic traits

Nested Analyses of Variance (ANOVA) were performed at the initial and final generations assayed for both 2010 and 2013 foundations to test for differences between samples from different years and locations. The mixed-effects linear model used was:

1 $$Y=\mu +Pop\{Year\ast Location\}+Year+Location+Year\ast Location+\varepsilon ,$$

where Y refers to the trait analysed, Pop{Year*Location} refers to the random factor Population nested in each combination of Year and Location (e.g. the three Ad replicate populations nested in the combination of Year 2010 and Location Adraga), with Year (2010 and 2013) and Location (Adraga and Groningen) as fixed factors. Planned pairwise comparisons between foundations assayed in synchrony were also performed (comparisons between the two recently introduced populations, as well as between each and the synchronously assayed TA populations). These were done in both the initial (5/6) and latest generations analysed (21/22 and 26/28). Type III sum of squares was used in all tests.

The evolutionary trajectory of each trait and foundation was analysed with a linear model defining Generation as a covariate, Population as a random factor, and also their interaction. To analyse the overall evolutionary response and differences in evolutionary rate between populations differing in the Year and Location of sampling, ANCOVA analyses were performed using the following mixed-effects linear model:

2 $$\begin{array}{c}Y={\rm{\mu }}+Pop\{Year\ast Location\}+Year+Location+Year\ast Location\\ \quad \,\,\,+Gen+Pop\{Year\ast Location\}\ast Gen+Year\ast Gen+Location\ast Gen\\ \quad \,\,\,+Year\ast Location\ast Gen+\varepsilon ,\end{array}$$

where Gen refers to the generations assayed (as covariate) and the other factors as above. As male body size data was not available at generation 28 of Ad and Gro, comparisons for this trait only included data obtained until generation 22 of these foundations, and generation 21 of PT and NL. Analyses defining body size data as a covariate were also done to account for its effects on other traits. Due to the unavailability of some male body size data (see above), when taking into account the effect of male size on male starvation resistance trajectories only data up to generations 21/22 was analysed.

All analyses were performed using differences to the mean values of the synchronously assayed control TA populations, to account for environmental variation between different assays.

We also analysed by linear regression the dependence of the evolutionary rate (slopes) on early differentiation across traits and populations. The slopes of each population*trait combination were computed using as data points for each generation assayed, the difference between each experimental population and the average of the synchronously assayed TA controls, divided by the latter. Early differentiation of each trait and population was estimated from the average trait values of generation 5 and 6 (2013 and 2010 data, respectively) also standardized to the average of the control. ANCOVAs were performed using the evolutionary rate as dependent variable, populations as random factors nested within foundations, and early differentiation as covariate. All data analyses described above were performed using STATISTICA 13 and EXCEL.

#### Chromosomal Inversions

Genetic differentiation in chromosomal inversion frequencies between source populations (Theta-p) was calculated at generations 1/2. Differentiation was also calculated between generations 1/2 and 23/25 for the different replicate populations of each foundation. Differentiation between foundations within generations 6/8 and 23/25 was estimated using a hierarchical AMOVA, with foundations and replicate populations nested within foundations (Theta-f). Theta-f and Theta-p values were obtained with GDA software^65^.

A principal component analysis (PCA) using a correlation matrix was applied to transformed inversion frequency data (2*square-root of inversion frequency) across chromosomal arrangements, populations and generations, following Balanyà *et al*.^41^.

To test for differences in temporal changes of specific inversions, ANOVAs were also performed on the arcsine transformed frequencies of the inversions with more than 5% of frequency across replicate populations. Comparisons between foundations with different location and year of sampling were performed on the slopes of the linear regression of the transformed frequencies of inversion across generations for each replicate population using the ANOVA model 1 defined above.

Significance of changes in frequency of specific arrangements (those with >5% in frequency across populations) between generations 6 and 25 for the 2010 data and between generations 8 and 23 for the 2013 data was assessed by Cochran-Mantel-Haenszel Chi-squared test (CMH statistic) to test for differences in replicated data^66^.

Simulations (9999 iterations) were performed on the same arrangements analysed with the CMH statistic, to test whether genetic drift could explain the temporal changes in inversion frequencies in each replicate population. Using generation 1 (for NL and PT) and 2 (for Ad and Gro) as starting points we simulated, using a multinomial distribution, the evolution of allele frequencies up to generations 23 and 25. A Wright-Fisher model was applied, assuming only drift and no mutation or migration. The effective population size (N_e_) estimates were obtained assuming an initial effective size of 10% of the census size and increments throughout generations reaching 30% by the last generations assayed, as done in ref. 35. *P*-values were defined as the proportion of times the simulated values were equal or higher than the observed ones (equal or lower in case of decreasing inversion frequencies) – see also ref. 35. False discovery rate (FDR) analysis was applied to account for multiple testing (ref. 67, theorem 1.3).

### Data Availability

The datasets generated during and/or analysed during the current study are available in the figshare repository, at doi:10.6084/m9.figshare.4797550.

## Electronic supplementary material

Supplementary Information
