# Supplementary material for: Predictable phenotypic, but not karyotypic, evolution of populations with contrasting initial history
Source: Sci Rep. 2017 Apr 19;7:913. doi: 10.1038/s41598-017-00968-1 (PMC5430419; doi:10.1038/s41598-017-00968-1)
Supplement: Supplementary file 1 — Supplementary Information [file 41598_2017_968_MOESM1_ESM.pdf]

Title: Predictable phenotypic, but not karyotypic, evolution of populations with contrasting initial history

Authors: Pedro Simões[1*], Inês Fragata[1#], Sofia G. Seabra[1], Gonçalo S. Faria[1£], Marta A. Santos[1§], Michael R. Rose [2], Mauro Santos[3] and Margarida Matos[1]

Affiliations:

[1] cE3c – Centre for Ecology, Evolution and Environmental Changes, Faculdade de Ciências, Universidade de Lisboa, Campo Grande, 1749-016 Lisboa, Portugal, Tel: +351 21 75 000 00

[2] Department of Ecology and Evolutionary Biology, University of California, Irvine, CA, USA.

[3] Departament de Genètica i de Microbiologia, Grup de Genòmica, Bioinformàtica i Biologia Evolutiva (GGBE), Universitat Autonòma de Barcelona, Spain.

[#] Current Address: Instituto Gulbenkian de Ciência, Oeiras, Portugal.

£ Current Address: University of Saint Andrews, School of Biology, Saint Andrews, Scotland, UK

§ Current Address: CEDOC – Centro de Estudos de Doenças Crónicas, Lisboa, Portugal.

[*]Correspondence should be addressed to: pmsimoes@fc.ul.pt

Table S1- Pairwise differences between synchronously assayed populations in the initial generation assayed (a, Ad vs Gro vs TA; PT vs NL vs TA), evolutionary rate (b, Ad vs Gro; PT vs NL) and final generations assayed (c and d, Ad vs Gro vs TA; PT vs NL vs TA)

a) At the first generation assayed

	Age of First Reproduction	Early Fecundity	Peak Fecundity	Female Starvation Resistance	Male Starvation Resistance	Female Size	Male Size
Ad vs Gro	$F_{1,4}$ = 5.331 m.s.	$F_{1,4}$ = 5.576 m.s.	$F_{1,4}$ = 5.626 m.s.	$F_{1,4}$ = 5.083 m.s.	$F_{1,4}$ = 6.440 m.s.	$F_{1,4.03}$ = 68.441 **	$F_{1,4.02}$ = 30.501 **
PT vs NL	$F_{1,4}$ = 0.000 n.s.	$F_{1,4}$ = 1.506 n.s.	$F_{1,4}$ = 2.805 n.s.	$F_{1,4}$ = 15.890 *	$F_{1,4}$ = 38.227 **	$F_{1,4.01}$ = 42.859 **	$F_{1,4.03}$ = 166.485 ***
Ad vs TA	$F_{1,4}$ = 16.874 *	$F_{1,4.01}$ = 183.506 ***	$F_{1,4.01}$ = 92.678 ***	$F_{1,4}$ = 0.0172 n.s.	$F_{1,4}$ = 2.836 n.s.	$F_{1,4}$ = 1.654 n.s.	$F_{1,4.01}$ = 3.160 n.s.
Gro vs TA	$F_{1,4}$ = 18.284 *	$F_{1,4}$ = 48.792 **	$F_{1,4}$ = 19.196 *	$F_{1,4}$ = 33.921 **	$F_{1,4}$ = 0.370 n.s.	$F_{1,4}$ = 38.233 **	$F_{1,4}$ = 50.650 **
PT vs TA	$F_{1,4}$ = 31.500 **	$F_{1,4}$ = 45.061 **	$F_{1,4.03}$ = 226.274 ***	$F_{1,4}$ = 0.002 n.s.	$F_{1,4}$ = 0.330 n.s.	$F_{1,4.02}$ = 0.069 n.s.	$F_{1,4.02}$ = 9.880 *
NL vs TA	$F_{1,4}$ = 3.802 n.s.	$F_{1,4}$ = 17.999 *	$F_{1,4.01}$ = 43.432 **	$F_{1,4}$ = 2.827 n.s.	$F_{1,4}$ = 4.653 m.s.	$F_{1,4.05}$ = 104.181 ***	$F_{1,4.03}$ = 47.683 **

Note: significance levels: p> 0.1 n.s.; 0.1>p>0.05 m.s.; 0.05>p>0.01*; 0.01>p>0.001**; p<0.001 ***. These analyses were performed at generation 6 of the 2010 foundations and generation 5 of the 2013 foundations

b) Evolutionary rate

	Age of First Reproduction	Early Fecundity	Peak Fecundity	Female Starvation Resistance	Male Starvation Resistance	Female Size	Male Size
Ad vs Gro	$F_{1,4}$ = 1.997 n.s.	$F_{1,4}$ = 3.685 n.s.	$F_{1,4.01}$ = 4.920 m.s.	$F_{1,4}$ = 3.138 n.s.	$F_{1,4}$ = 1.405 n.s.	$F_{1,4.01}$ = 5.346 m.s.	$F_{1,4}$ = 6.195 m.s.
PT vs NL	$F_{1,4}$ = 0.204 n.s.	$F_{1,4}$ = 0.026 n.s.	$F_{1,4.01}$ = 0.012 n.s.	$F_{1,4}$ = 0.610 n.s.	$F_{1,4}$ = 1.654 n.s.	$F_{1,4}$ = 0.120 n.s.	$F_{1,4}$ = 0.101 n.s.

c) At the last generation assayed

	Age of First Reproduction	Early Fecundity	Peak Fecundity	Female Starvation Resistance	Male Starvation Resistance	Female Size	Male Size
Ad vs Gro	$F_{1,4} = 0.117$ n.s.	$F_{1,4.01} = 0.108$ n.s.	$F_{1,4} = 0.001$ n.s.	$F_{1,4} = 0.096$ n.s.	$F_{1,4.01} = 0.086$ n.s.	$F_{1,4.01} = 1.362$ n.s.	$F_{1,4} = 0.711$ n.s.
PT vs NL	$F_{1,4.01} = 0.017$ n.s.	$F_{1,4} = 0.278$ n.s.	$F_{1,4.01} = 0.306$ n.s.	$F_{1,4.01} = 0.155$ n.s.	$F_{1,4.01} = 0.761$ n.s.	$F_{1,4.03} = 9.685$ *	$F_{1,4.01} = 9.058$ *
Ad vs TA	$F_{1,4} = 0.392$ n.s.	$F_{1,4} = 21.538$ **	$F_{1,4} = 5.665$ m.s.	$F_{1,4} = 0.966$ n.s.	$F_{1,4.01} = 1.947$ n.s.	$F_{1,4} = 2.442$ n.s.	$F_{1,4} = 18.636$ *
Gro vs TA	$F_{1,4} = 0.058$ n.s.	$F_{1,4} = 16.726$ *	$F_{1,4} = 3.491$ n.s.	$F_{1,4} = 0.643$ n.s.	$F_{1,4} = 1.939$ n.s.	$F_{1,4.01} = 18.376$ *	$F_{1,4} = 8.570$ *
PT vs TA	$F_{1,4.01} = 2.015$ n.s.	$F_{1,4.01} = 4.755$ m.s.	$F_{1,4.02} = 1.558$ n.s.	$F_{1,4.05} = 1.316$ n.s.	$F_{1,4.04} = 0.001$ n.s.	$F_{1,4} = 0.640$ n.s.	$F_{1,4.01} = 1.210$ n.s.
NL vs TA	$F_{1,4.03} = 3.931$ n.s.	$F_{1,4.02} = 13.585$ *	$F_{1,4.05} = 2.164$ n.s.	$F_{1,4.03} = 0.562$ n.s.	$F_{1,4.02} = 0.884$ n.s.	$F_{1,4.03} = 6.490$ m.s.	$F_{1,4} = 7.250$ m.s.

d) At generation 21/22

	Age of First Reproduction	Early Fecundity	Peak Fecundity	Female Starvation Resistance	Male Starvation Resistance	Female Size	Male Size
Ad vs Gro	$F_{1,4.01} = 1.828$ n.s.	$F_{1,4} = 0.483$ n.s.	$F_{1,4} = 0.395$ n.s.	$F_{1,4} = 0.186$ n.s.	$F_{1,4} = 1.871$ n.s.	$F_{1,4} = 0.279$ n.s.	$F_{1,4} = 0.711$ n.s.
PT vs NL	$F_{1,4} = 0.001$ n.s.	$F_{1,4} = 0.091$ n.s.	$F_{1,4} = 0.002$ n.s.	$F_{1,4.01} = 6.056$ m.s.	$F_{1,4} = 0.593$ n.s.	$F_{1,4} = 4.350$ n.s.	$F_{1,4.03} = 9.791$ *
Ad vs TA	$F_{1,4.01} = 0.487$ n.s.	$F_{1,4} = 4.008$ n.s.	$F_{1,4} = 2.712$ n.s.	$F_{1,4} = 0.0004$ n.s.	$F_{1,4} = 0.092$ n.s.	$F_{1,4.01} = 7.650$ m.s.	$F_{1,4} = 18.636$ *
Gro vs TA	$F_{1,4.01} = 0.248$ n.s.	$F_{1,4} = 4.000$ n.s.	$F_{1,4} = 4.605$ m.s.	$F_{1,4.01} = 0.442$ n.s.	$F_{1,4.01} = 4.216$ n.s.	$F_{1,4} = 7.920$ *	$F_{1,4} = 8.570$ *
PT vs TA	$F_{1,4} = 1.422$ n.s.	$F_{1,4} = 4.609$ m.s.	$F_{1,4} = 5.469$ m.s.	$F_{1,4} = 1.743$ n.s.	$F_{1,4} = 0.964$ n.s.	$F_{1,4.01} = 0.610$ n.s.	$F_{1,4.03} = 1.659$ n.s.
NL vs TA	$F_{1,4} = 0.838$ n.s.	$F_{1,4} = 41.579$ **	$F_{1,4} = 16.432$ *	$F_{1,4} = 0.919$ n.s.	$F_{1,4} = 0.064$ n.s.	$F_{1,4.01} = 3.770$ n.s.	$F_{1,4.03} = 46.819$ **

Note: significance levels: $p > 0.1$ n.s.; $0.1 > p > 0.05$ m.s.; $0.05 > p > 0.01$*; $0.01 > p > 0.001$**; $p < 0.001$ ***.
For Male size in the 2010 populations generation 21 is the last generation with available data (see material and methods). It is thus presented in both c) and d).

Table S2 - Effect of body size on differences in early differentiation (a) and evolutionary rate (b) in phenotypic traits (ANCOVA) across generations.

a) Early Differentiation

Model parameters	Age of First Reproduction	Early Fecundity	Peak Fecundity	Female Starvation Resistance	Male Starvation Resistance
Year	$F_{1,8.21}$ = 11.309 **	$F_{1,8.10}$ = 4.212 m.s.	$F_{1,8.10}$ = 2.275 n.s.	$F_{1,8.09}$ = 0.741 n.s.	$F_{1,8.02}$ = 6.823 *
Location	$F_{1,14.91}$ = 0.059 n.s.	$F_{1,11.02}$ = 0.425 n.s.	$F_{1,11.08}$ = 1.418 n.s.	$F_{1,10.86}$ = 4.654 m.s.	$F_{1,11.24}$ = 10.786 **
Year*Location	$F_{1,8.84}$ = 1.933 n.s.	$F_{1,8.39}$ = 0.023 n.s.	$F_{1,8.40}$ = 0.284 n.s.	$F_{1,8.37}$ = 0.643 n.s.	$F_{1,8.15}$ = 1.445 n.s.
CS	$F_{1,256}$ = 7.790 **	$F_{1,256}$ = 16.121 ***	$F_{1,256}$ = 9.138 **	$F_{1,255}$ = 4.286 *	$F_{1,248}$ = 10.869 **

b) Evolutionary rate

Model parameters	Age of First Reproduction	Early Fecundity	Peak Fecundity	Female Starvation Resistance	Male Starvation Resistance	Male Starvation Resistance ł
Gen	$F_{1,8.08}$ = 14.230 **	$F_{1,8.03}$ = 58.092 ***	$F_{1,8.05}$ = 76.396 ***	$F_{1,8.05}$ = 8.533 *	$F_{1,8.13}$ = 0.925 n.s.	$F_{1,8.01}$ = 0.376 n.s.
Year*Gen	$F_{1,8.17}$ = 4.830 m.s.	$F_{1,8.06}$ = 0.375 n.s.	$F_{1,8.08}$ = 0.001 n.s.	$F_{1,8.09}$ = 5.902 *	$F_{1,8.01}$ = 6.633 *	$F_{1,8.01}$ = 7.429 *
Location*Gen	$F_{1,8.17}$ = 0.065 n.s.	$F_{1,8.07}$ = 0.388 n.s.	$F_{1,8.11}$ = 0.533 n.s.	$F_{1,8.12}$ = 3.547 m.s.	$F_{1,8.10}$ = 3.665 m.s.	$F_{1,8.01}$ = 4.879 m.s.
Year*Location*Gen	$F_{1,8.25}$ = 0.261 n.s.	$F_{1,8.10}$ = 0.071 n.s.	$F_{1,8.16}$ = 0.734 n.s.	$F_{1,8.18}$ = 1.002 n.s.	$F_{1,8.06}$ = 1.603 n.s.	$F_{1,8.01}$ = 0.368 n.s.
CS	$F_{1,1180}$ = 26.355 ***	$F_{1,1181}$ = 56.246 ***	$F_{1,1173}$ = 27.731 ***	$F_{1,1172}$ = 1.786 n.s.	$F_{1,1007}$ = 28.051 ***	-

Note: significance levels: p> 0.1 n.s.; 0.1>p>0.05 m.s.; 0.05>p>0.01*; 0.01>p>0.001**; p<0.001 ***. Body size was defined as covariate.

Analyses of the Evolutionary rate of male starvation resistance used data up to generations 21 of NL/PT and 22 of Gro/Ad.

ł analysis with data up to generations 21/22 but without defining male body size as covariate

Table S3 - Analyses of the evolutionary response (ANCOVA) for each foundation and trait

Foundation	Age of First Reproduction	Early Fecundity	Peak Fecundity	Female Starvation Resistance	Male Starvation Resistance	Female Size	Male Size
Ad	$F_{1,2}$ = 17.283 m.s.	$F_{1,2}$ = 109.528 **	$F_{1,2}$ = 57.064 *	$F_{1,2}$ = 0.519 n.s.	$F_{1,2}$ = 22.993 *	$F_{1,2}$ = 6.247 n.s.	$F_{1,2}$ = 66.912 *
Gro	$F_{1,2}$ = 68.183 *	$F_{1,2}$ = 27.766 *	$F_{1,2}$ = 31.075 *	$F_{1,2}$ = 10.682 m.s.	$F_{1,2}$ = 0.281 n.s.	$F_{1,2}$ = 0.843 n.s.	$F_{1,2}$ = 0.239 n.s.
PT	$F_{1,2}$ = 0.144 n.s.	$F_{1,2}$ = 10.731 m.s.	$F_{1,2}$ = 13.683 m.s.	$F_{1,2}$ = 0.001 n.s.	$F_{1,2}$ = 1.562 n.s.	$F_{1,2}$ = 0.814 n.s.	$F_{1,2}$ = 1.483 n.s.
NL	$F_{1,2}$ = 0.544 n.s.	$F_{1,2}$ = 25.830 *	$F_{1,2.07}$ = 2034.0 ***	$F_{1,2}$ = 1.367 n.s.	$F_{1,2}$ = 0.984 n.s.	$F_{1,2}$ = 0.189 n.s.	$F_{1,2}$ = 0.603 n.s.

Note: significance levels: p> 0.1 n.s.; 0.1>p>0.05 m.s.; 0.05>p>0.01*; 0.01>p>0.001**; p<0.001 ***.

Table S4 - Analyses of differences (ANOVA) in phenotypic traits at the last generation assayed

Model parameters	Age of First Reproduction	Early Fecundity	Peak Fecundity	Female Starvation Resistance	Male Starvation Resistance	Female Size	Male Size
Year	$F_{1,8.02}$ = 0.771 n.s.	$F_{1,8.02}$ = 0.117 n.s.	$F_{1,8.04}$ = 1.046 n.s.	$F_{1,8.04}$ = 4.365 m.s.	$F_{1,8.07}$ = 1.093 n.s.	$F_{1,8.06}$ = 2.858 n.s.	$F_{1,8.03}$ = 1.098 n.s.
Location	$F_{1,8.02}$ = 0.121 n.s.	$F_{1,8.02}$ = 0.370 n.s.	$F_{1,8.04}$ = 0.186 n.s.	$F_{1,8.04}$ = 0.209 n.s.	$F_{1,8.07}$ = 0.854 n.s.	$F_{1,8.06}$ = 9.975 *	$F_{1,8.03}$ = 6.567 *
Year*Location	$F_{1,8.02}$ = 0.035 n.s.	$F_{1,8.02}$ = 0.175 n.s.	$F_{1,8.04}$ = 0.227 n.s.	$F_{1,8.04}$ = 0.004 n.s.	$F_{1,8.07}$ = 0.422 n.s.	$F_{1,8.06}$ = 2.749 n.s.	$F_{1,8.03}$ = 1.498 n.s.
Pop(Year*Location)	$F_{8,196}$ = 0.035 n.s.	$F_{8,195}$ = 2.619 **	$F_{8,185}$ = 2.789 **	$F_{8,184}$ = 2.806 **	$F_{8,183}$ = 1.348 n.s.	$F_{8,164}$ = 3.333 **	$F_{8,246}$ = 3.192 **

Note: significance levels: p> 0.1 n.s.; 0.1>p>0.05 m.s.; 0.05>p>0.01*; 0.01>p>0.001**; p<0.001 ***.
These analyses were performed at generation 28 (generation 22 for Male Size) of the 2010 foundations and generation 26 (generation 21 for Male Size) of the 2013 foundations.

Table S5 - Analyses of the genetic differentiation in inversion frequencies between foundations and generations.

a) Genetic differentiation in the different generations

	Comparison	Theta	CI lower	CI upper
Ad vs Gro	Ad_G2 vs Gro_G2	0.302	0.199	0.395
	Ad_G6 vs Gro_G6	0.289	0.178	0.381
	Ad_G25 vs Gro_G25	0.269	0.168	0.357
PT vs NL	PT_G1 vs NL_G1	0.332	0.244	0.415
	PT_G8 vs NL_G8	0.292	0.173	0.396
	PT_G23 vs NL_G23	0.122	0.077	0.170
Ad vs PT	Ad_G2 vs PT_G1	-0.004	-0.007	-0.003
	Ad_G6 vs PT_G8	0.017	-0.004	0.031
	Ad_G25 vs PT_G23	0.135	0.074	0.176
Gro vs NL	Gro_G2 vs NL_G1	-0.002	-0.006	0.005
	Gro_G6 vs NL_G8	0.027	0.005	0.046
	Gro_G25 vs NL_G23	0.184	0.114	0.262

Note**:** Upper and lower Confidence Intervals (95%) were obtained after 5000 bootstrap iterations across chromosomes. Theta-p (differentiation between populations) were calculated at generations 1 and 2 and in comparisons between generations 1/2 and 23/25.

Theta-f (hierarchical AMOVA analysis with foundations and replicate populations) were computed at generations 6/8 and at generations 23/25.

Table S5 - Analyses of the genetic differentiation in inversion frequencies between foundations and generations.

b) Genetic differentiation across initial and final generations for each foundation

Comparison	Theta-p	CI lower	CI upper
PT_G1 vs PT1_G23	0.287	0.190	0.342
PT_G1 vs PT2_G23	0.125	0.065	0.170
PT_G1 vs PT3_G23	0.174	0.065	0.258
NL_G1 vs NL1_G23	0.166	0.097	0.215
NL_G1 vs NL2_G23	0.197	0.097	0.268
NL_G1 vs NL3_G23	0.314	0.186	0.428
Ad_G2 vs Ad1_G25	0.111	0.007	0.208
Ad_G2 vs Ad2_G25	0.163	0.067	0.234
Ad_G2 vs Ad3_G25	0.169	0.046	0.336
Gro_G2 vs Gro1_G25	0.174	0.061	0.314
Gro_G2 vs Gro2_G25	0.110	0.021	0.235
Gro_G2 vs Gro3_G25	0.134	0.071	0.188

Note**:** Upper and lower Confidence Intervals (95%) were obtained after 5000 bootstrap iterations across chromosomes. Theta-p (differentiation between populations) were calculated at generations 1 and 2 and in comparisons between generations 1/2 and 23/25.

Table S6 - ANOVA Analyses on the slopes of the transformed frequencies of specific arrangements across generations.
A) For arrangements present in both locations; B) For arrangements present only in Adraga (E_{1+2+9} and O_{3+4+7}) or Groningen (A_1 and U_{ST})

A)

Model Parameters	A_2	A_{ST}	E_{1+2}	$E_{1+2+9+12}$	E_{ST}	J_{ST} (or J_1)	O_{3+4}	O_{3+4+8}	O_{ST}	U_{1+2}	U_{1+2+8}
Intercept	58.071 ***	43.172 ***	10.573 *	65.250 ***	43.052 ***	96.062 ***	12.983 **	4.122 n.s.	7.996 *	10.042 *	57.204 ***
Location	5.709 *	2.692 n.s.	8.332 *	0.102 n.s.	6.192 *	5.474 *	7.069 *	0.235 n.s.	7.911 *	7.248 *	0.083 n.s.
Year	0.766 n.s.	0.297 n.s.	3.821 m.s.	7.479 *	3.337	0.001 n.s.	3.036 n.s.	0.338 n.s.	0.323 n.s.	11.845 **	37.523 ***
Year*Location	1.166 n.s.	0.668 n.s.	0.036 n.s.	0.098 n.s.	0.889 n.s.	1.669 n.s.	5.022 m.s.	4.679 m.s.	1.106 n.s.	0.646 n.s.	7.556 *

Note: significance levels: p> 0.1 n.s.; 0.1>p>0.05 m.s.; 0.05>p>0.01*; 0.01>p>0.001**; p<0.001 ***.
For all analyses the degrees of freedom of the effect and the error term were respectively 1 and 8.

B)

Model Parameters	A_1	E_{1+2+9}	O_{3+4+7}	U_{ST}
Intercept	0.554 n.s.	25.582 **	165.88 ***	13.893 *
Year (Adraga)	-	0.588 n.s.	3.884 n.s.	-
Year (Groningen)	0.099 n.s.	-	-	2.608 n.s.

Note: significance levels: p> 0.1 n.s.; 0.1>p>0.05 m.s.; 0.05>p>0.01*; 0.01>p>0.001**; p<0.001 ***.
For all analyses the degrees of freedom of the effect and the error term were respectively 1 and 4.

Table S7 - Cochran-Mantel-Haenszel (CMH) statistic and simulations (for each replicate population) applied to the most frequent arrangements.

Arrangement	Ad_1	Ad_2	Ad_3	Ad (CMH)	Gro_1	Gro_2	Gro_3	Gro (CMH)	PT_1	PT_2	PT_3	PT (CMH)	NL_1	NL_2	NL_3	NL (CMH)
A_2	n.s.	n.s.	n.s.	15.686***	m.s.	n.s.	n.s.	53.020 ***	n.s.	n.s.	n.s.	3.012 n.s.	*	m.s.	***	40.807 ***
A_{ST}	---	---	---		m.s.	n.s.	n.s.	44.318 ***	---	---	---		n.s.	*	*	32.333 ***
A_1					n.s.	n.s.	n.s.	0.175 n.s.					n.s.	n.s.	n.s.	0.699 n.s.
E_{ST}	n.s.	n.s.	n.s.	13.295 ***	n.s.	n.s.	n.s.	29.365 ***	n.s.	n.s.	n.s.	20.698 ***	*	n.s.	**	38.577 ***
$E_{1+2+9+12}$	n.s.	*	n.s.	69.651 ***	n.s.	n.s.	*	30.592 ***	***	*	*	83.112 ***	**	*	***	102.84 ***
E_{1+2}	n.s.	n.s.	n.s.	3.735 n.s.	n.s.	n.s.	n.s.	0.945 n.s.	m.s.	n.s.	n.s.	12.339 **	n.s.	n.s.	n.s.	12.240 **
E_{1+2+9}	n.s.	n.s.	n.s.	48.553 ***					m.s.	n.s.	n.s.	26.194 ***				
$E_{1+2+9+3}$	n.s.	n.s.	n.s.	9.301 **												
J_{ST} (or J_1)	n.s.	n.s.	n.s.	23.237 ***	**	*	n.s.	99.474 ***	n.s.	n.s.	n.s.	24.096 ***	n.s.	n.s.	*	31.787 ***
O_{ST}	**	n.s.	n.s.	61.469 ***	n.s.	n.s.	n.s.	2.477 n.s.	*	m.s.	n.s.	40.708 ***	n.s.	n.s.	n.s.	15.220 ***
O_{3+4}	n.s.	*	***	36.983 ***	n.s.	n.s.	n.s.	16.430 ***	n.s.	n.s.	*	21.820 ***	n.s.	n.s.	m.s.	28.457 ***
O_{3+4+7}	*	*	**	102.15 ***					**	**	***	79.774 ***				
O_{3+4+8}	n.s.	n.s.	n.s.	15.449 ***	n.s.	n.s.	n.s.	0.742 n.s.	n.s.	n.s.	n.s.	3.818 n.s.	n.s.	n.s.	n.s.	2.037 n.s.
U_{1+2}	---	---	---		n.s.	n.s.	n.s.	3.464 n.s.	---	---	---		n.s.	n.s.	n.s.	14.781 ***
U_{1+2+8}	n.s.	n.s.	n.s.	14.071 ***	n.s.	n.s.	n.s.	2.112 n.s.	**	n.s.	n.s.	28.697 ***	**	**	*	80.993 ***
U_{ST}					n.s.	n.s.	n.s.	7.747 *					m.s.	*	n.s.	29.917 ***

Note: CMH Statistic and significance levels are presented for each foundation (pooled ratio); Significance of the simulations applied (see also material and methods) is shown for each replicate population.

Significance levels after FDR correction:

For Ad (n=12): *** $p<0.00032$ ($\alpha=0.001$); ** $0.00032<p<0.0032$ ($\alpha=0.01$); * $0.0032<p<0.016$ ($\alpha=0.05$); m.s. $0.016<p<0.032$ ($\alpha=0.1$); n.s. $p>0.032$.

For PT (n=11): *** $p<0.00033$ ($\alpha=0.001$); ** $0.00033<p<0.0033$ ($\alpha=0.01$); * $0.0033<p<0.017$ ($\alpha=0.05$); m.s. $0.017<p<0.033$ ($\alpha=0.1$); n.s. $p>0.033$.

For Gro and NL (n=13): *** $p<0.00031$ ($\alpha=0.001$); ** $0.00031<p<0.0031$ ($\alpha=0.01$); * $0.0031<p<0.016$ ($\alpha=0.05$); m.s. $0.016<p<0.031$ ($\alpha=0.1$); n.s. $p>0.031$.

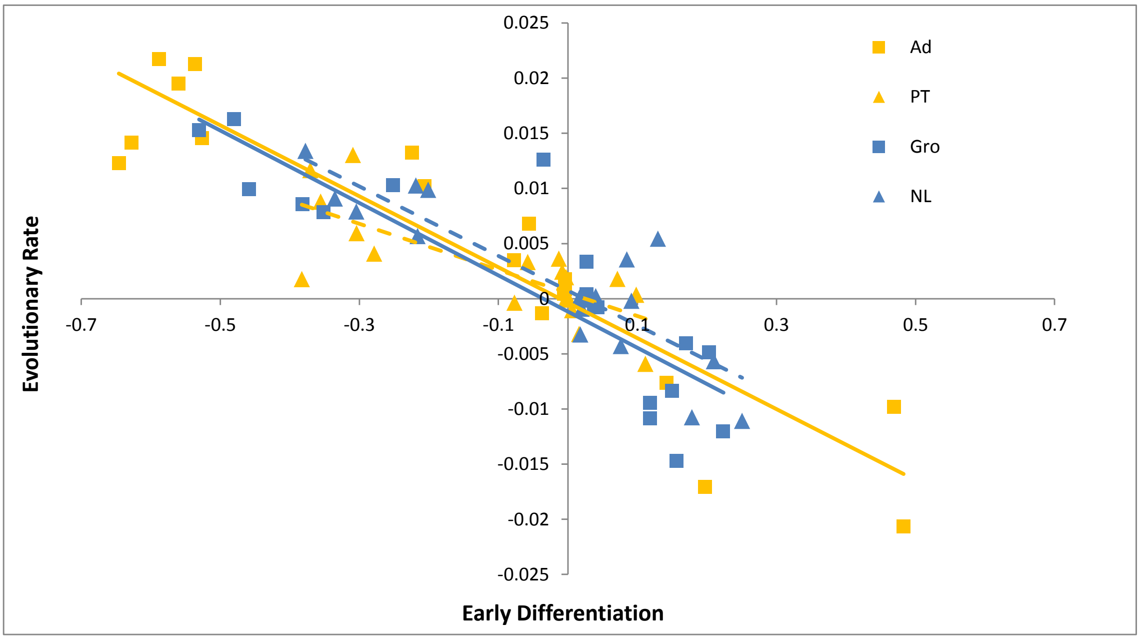

Figure S1 - Evolutionary rate as a function of early differentiation for phenotypic traits.

Populations from Portugal (Ad and PT) – Orange; Populations from Netherlands (Gro and NL) – Blue; 2010 Populations (Ad and Gro) - Squares, solid line; 2013 Populations (PT and NL) – Triangles, dashed line. The dependence of evolutionary slope on early differentiation was highly significant (ANCOVA $F_{1,11.5} = 100.54$, $P<0.0001$). All regression models are highly significant ($P<0.001$). The slopes of these regression models were not significantly different across foundations (ANCOVA $F_{3,9.5} = 0.817$, $P<0.52$).

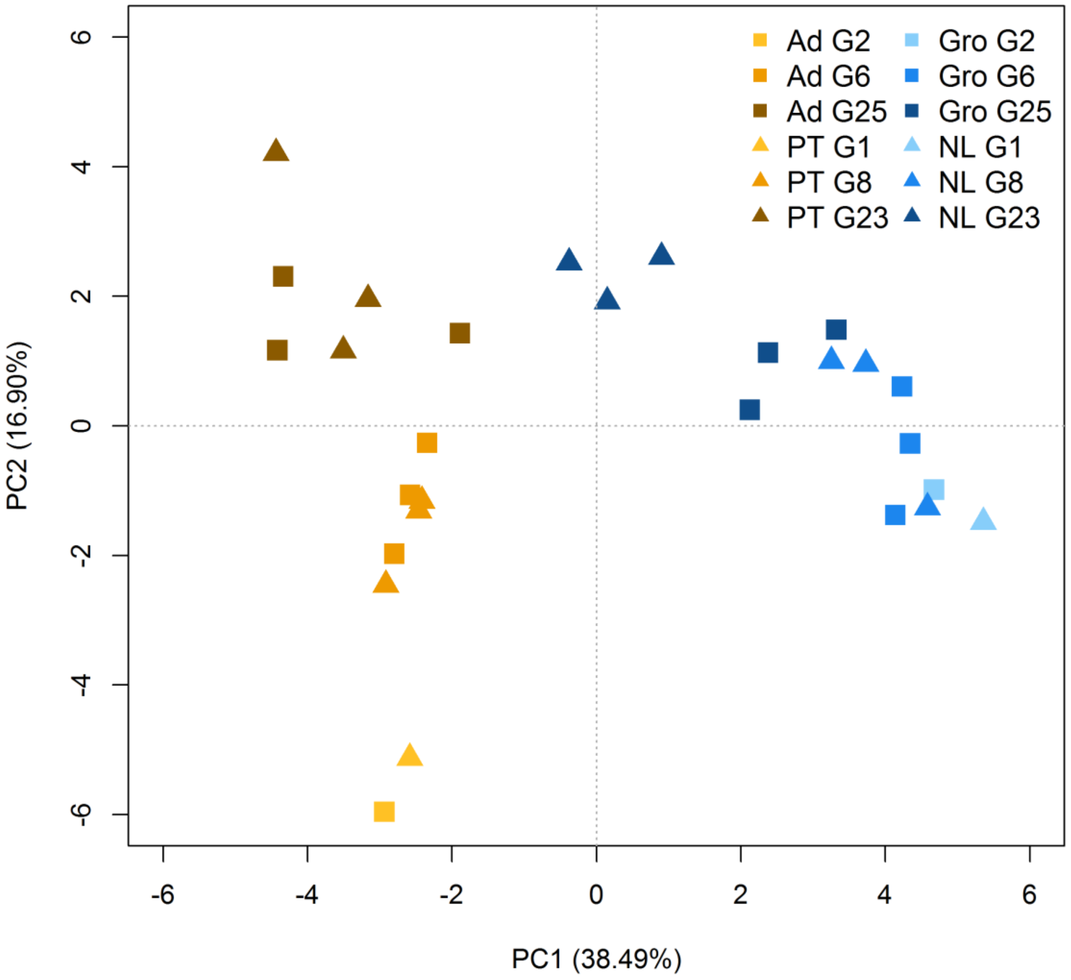

Figure S2 - Principal component analysis (PCA) for chromosomal inversion frequencies across generations and foundations.

Populations from Portugal (Ad and PT) – Orange; Populations from Netherlands (Gro and NL) – Blue; 2010. Populations (Ad and Gro) - Squares; 2013 Populations (PT and NL) – Triangles.

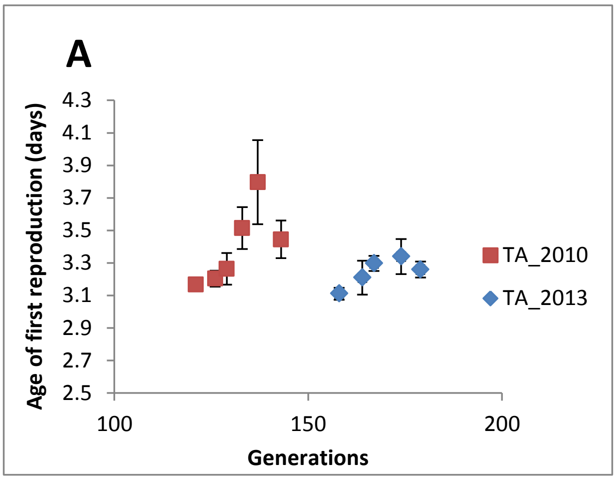

Fig. S3A – Age of first reproduction

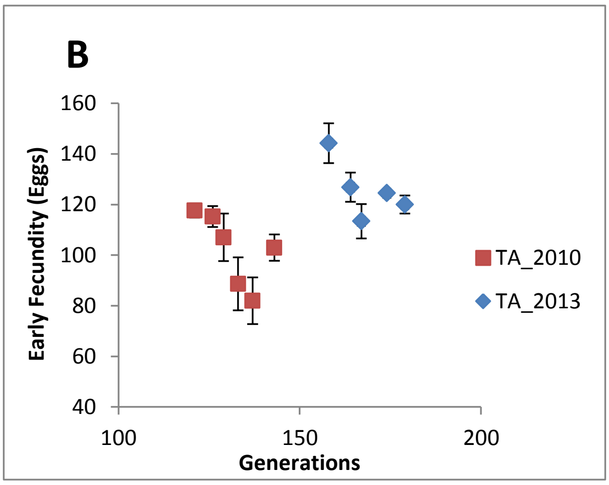

Fig. S3B – Early Fecundity

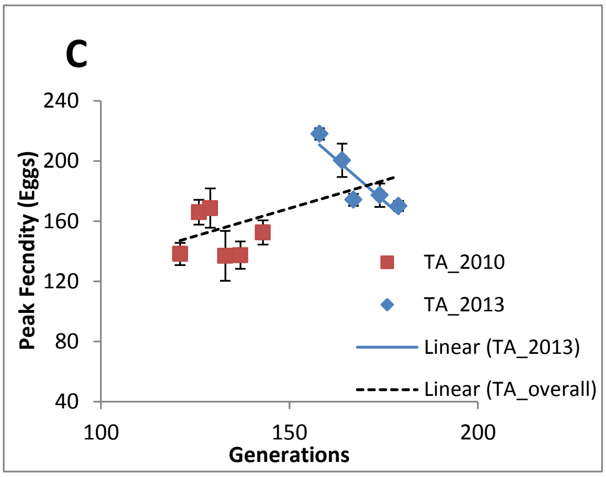

Fig. S3C – Peak Fecundity

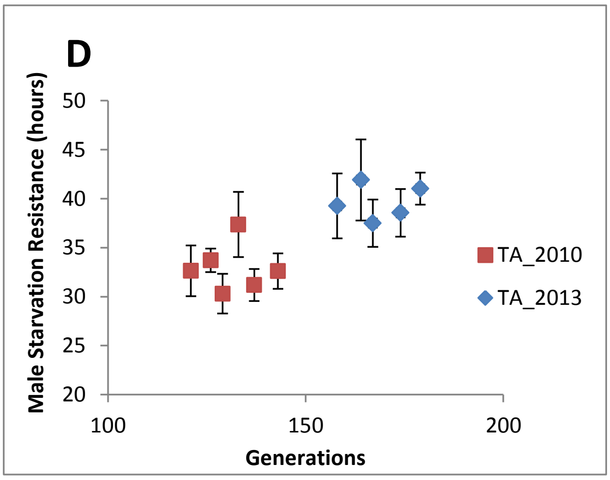

Fig. S3D – Male Starvation Resistance

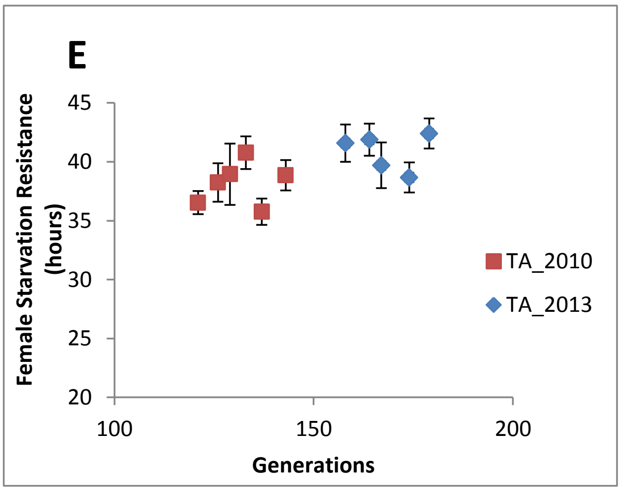

Fig. S3E – Female Starvation Resistance

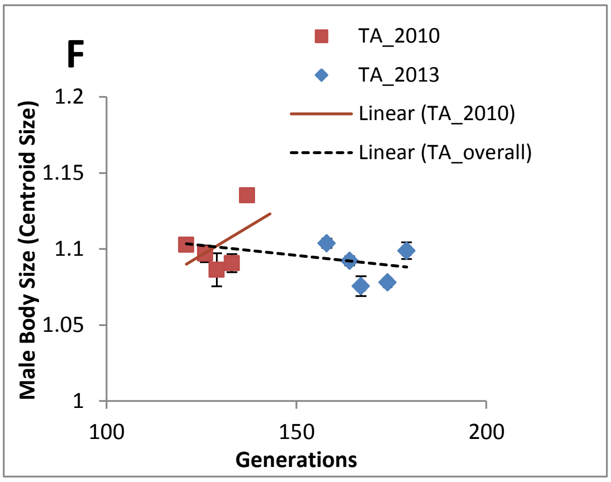

Fig. S3F – Male Body Size

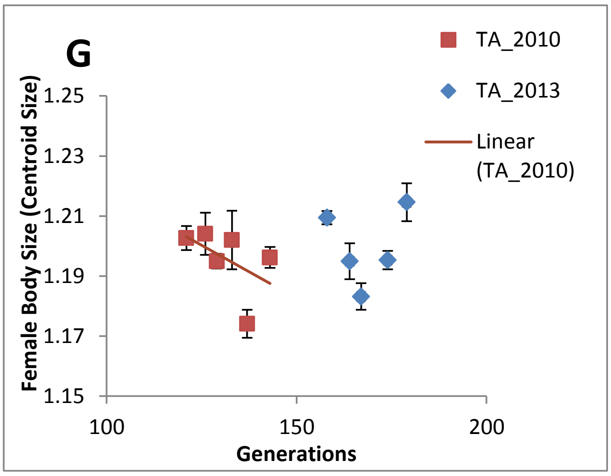

Fig. S3G – Female Body Size

Figure S3 – Temporal changes of the TA control populations in each period (2010 and 2013) studied for the seven phenotypic traits studied. Traits: Age of first reproduction (A), Early (B) and Peak (C) Fecundity, Male (D) and Female (E) Starvation Resistance, Male (F) and Female (G) Body Size.

For most traits there were no significant linear trends, with the exception of peak fecundity for the 2013 assays (p=0.022) and body size for the 2010 period (females: p=0.041; males: p=0.044). Moreover across the two periods only peak fecundity and male body size showed a significant trend (dashed lines; p=0.022; p=0.033, respectively). It is important to point out that there were clear discrepancies between the overall and partial trends in both these traits. The trend detected for peak fecundity in the 2013 assays was negative, while the overall trend including all assays was slightly positive. Male body size showed a significant positive trend in the 2010 assays but a general negative one across the two periods. These trends are likely caused by non-genetic, micro-environmental changes between assays.
